# Re-challenge with pemetrexed in advanced mesothelioma: a multi-institutional experience

**DOI:** 10.1186/1756-0500-5-482

**Published:** 2012-09-03

**Authors:** Alessandra Bearz, Renato Talamini, Gilda Rossoni, Antonio Santo, Vincenzo de Pangher, Gianpiero Fasola, Francesco Rosetti, Adolfo Favaretto, Vanesa Gregorc, Massimiliano Berretta, Sandra Santarossa, Eleonora Berto, Umberto Tirelli

**Affiliations:** 1 Medical Oncology, IRCCS, Aviano, PN, Italy; 2 Unit of Epidemiology and Biostatistics, IRCCS, Aviano, PN, Italy; 3 Medical Oncology, IRCCS-S Raffaele, Milan, Italy; 4 General Hospital, GIVOV, Verona, Italy; 5 General Hospital, Gorizia, Italy; 6 General Hospital Udine, Udine, Italy; 7 General Hospital, Mirano, Italy; 8 IOV Padova, Padova, Italy; 9 National Cancer Institute of Aviano, Via Franco Gallini 2, Aviano, PN, 33081, Italy

## Abstract

**Background:**

Although first-line therapy for patients affected by advanced mesothelioma is well established, there is a lack of data regarding the impact of second-line treatment.

**Methods:**

We retrospectively collected data of patients affected by advanced mesothelioma, already treated with first-line therapy based on pemetrexed and platin, with a response (partial response or stable disease) lasting at least 6 months, and re-treated with a pemetrexed-based therapy at progression. The primary objective was to describe time to progression and overall survival after re-treatment.

**Results:**

Overall across several Italian oncological Institutions we found 30 patients affected by advanced mesothelioma, in progression after a 6-month lasting clinical benefit following a first-line treatment with cisplatin and pemetrexed, and re-challenged with a pemetrexed-based therapy. In these patients we found a disease control rate of 66%, with reduction of pain in 43% of patients. Overall time to progression and survival were promising for a second-line setting of patients with advanced mesothelioma, being 5.1 and 13.6 months, respectively.

**Conclusions:**

In our opinion, when a patient has a long-lasting benefit from previous treatment with pemetrexed combined with a platin compound, the same treatment should be offered at progression.

## Background

The current standard of care in the management of advanced malignant pleural mesothelioma is a platinum-based chemotherapy [1]; a number of phase II trials have suggested an activity for cisplatin and gemcitabine combination [2,3], while the landmark trial is the one by Vogelzang and Colleagues comparing cisplatin plus pemetrexed to cisplatin alone, demonstrating a survival benefit for the combination of 2.8 months versus cisplatin alone [4]. A subsequent trial showed a 2.6-month improvement in overall survival (OS) for the combination with cisplatin and raltitrexed versus cisplatin alone, 11.4 versus 8.8 months, respectively [5]. Although carboplatin has been shown to be less effective than cisplatin in other malignancies like Non Small-Cell Lung Cancer (NSCLC) [6], when cisplatin is contraindicated for comorbidities, it may be replaced by carboplatin; moreover several phase I and II trials where carboplatin was combined with pemetrexed or gemcitabine in malignant mesothelioma have given interesting results [7-9]. Due to similar histologic features, treatment for peritoneal mesothelioma has followed the same recommendations [10,11].

No activity for second-line treatment has been clearly reported in patients affected by malignant pleural mesothelioma; pemetrexed alone or combined with platin has shown efficacy even in patients previously treated with other drugs than pemetrexed [12,13], and some activity has been demonstrated for vinorelbine [14].

In absence of a standard second-line treatment for malignant mesothelioma, we hypothesized efficacy from re-treatment with pemetrexed alone or in combination with platin, when time to progression (TTP), calculated from the end of first-line chemotherapy and radiologic evidence of progression, was greater or equal than 6 months, as suggested for other pathologies with few therapeutic options [15]. We performed a retrospective analysis of patients re-treated with pemetrexed-based therapy in second line, to assess our hypothesis.

## Methods

We retrospectively collected patients affected by mesothelioma from several Italian Institutions (IRCCS-Aviano, IRCCS-S. Raffaele Milan, and General Hospitals of Verona, Gorizia, Udine, Mirano, and Padova), after obtaining approval from IRCCS – Aviano internal ethics committee. In all these Institutions there was the policy to re-treat all patients affected by advanced malignant mesothelioma with the same drugs used in first line, when certain features were observed. Patients had to be treated with a pemetrexed-platin combination as first-line chemotherapy, obtaining partial response (PR) or stable disease (SD), with a further TTP greater or equal than 6 months. We analyzed characteristics and outcome only of those patients treated again with a pemetrexed-based therapy after progression.

The regimens administered as re-treatment were pemetrexed at a dose of 500 mg/m^2^ combined with a platinum compound (cisplatin at a dose of 75 mg/m^2^ or carboplatin at an area under the plasma concentration-time curve –AUC - of 5 mg/ml/min) every three weeks or pemetrexed single agent at a dose of 500 mg/m^2^ every three weeks, using standard vitamin supplementation and dexamethasone prophylaxis. All the patients had a calculated creatinine clearance value (according to Cockcroft and Gault formula [16]) greater than 45 mL/min, and good hepatic and bone marrow activity. Before re-treatment, all the patients were studied with a chest and abdomen CT scan, repeated every two cycles. Responses were evaluated through RECIST modified criteria for mesothelioma [17]. Information on survival was obtained through an active follow-up based on verification of vital status of the patients, and survival analysis was measured from the date of the first pemetrexed-based re-treatment to death. If survival status was unknown at the final follow-up, OS time was censored at the last contact date. TTP was measured from the beginning of second line of therapy to second relapse. OS and TTP analyses were computed by the Kaplan-Meier method [18], and log-rank test was used to test the difference between subgroups of treatment. In all cases, statistical significant was claimed for ≤0.05.

## Results

Between January 2005 and December 2009 we collected 30 patients from 7 Italian Institutions; their clinical characteristics are reported in Table 1. The majority of all patients were male (77%); with an ECOG Performance Status (PS) of 1 in 15 patients (50%) and 0 in 15 (50%); median age at diagnosis was 64.1 years. Sixteen patients (53%) had a history of exposition to asbestos, while it was unknown or unclear for all the others. Regarding histology, 28 patients (93%) were affected by epithelioid mesothelioma, and 2 (7%) by mixed. At diagnosis, stage IV mesothelioma, according to IMIG classification, was diagnosed in 12 out of 30 patients (40%), stage III in 8 (27%), stage II in 9 (30%), and one patient (3%) had an extrapleural localization (tunical vaginalis). Nine patients (30%) never underwent surgery; while all the other had major surgery, in particular, 11 patients (37%) had palliative pleural decortication; 6 (20%) extrapleural pneumonectomy; 3 (10%) videothoracoscopy and pleurodesis, and 1 (3%) orchiectomy. Time from surgery to the beginning of treatment with systemic chemotherapy varied between 1 and 30 months; most of the patients received chemotherapy just after recovery from surgery; median time between surgery and the beginning of chemotherapy was 6.5 months. Only patients with a tumor assessable before the administration of first line cisplatin and pemetrexed were included in the analysis.

**Table 1:** Characteristics of 30 patients with malignant mesothelioma and retreated with pemetrexed-based therapy as second-line therapy

	**N. (%)**
**N. of patients**	30 (100)
**Histology**:	
---Epidermoid	28 (93)
---Mixed	2 (7)
**Previous surgery**:	
---Palliative pleural decortications	11 (37)
---Extrapleural pneumonectomy	6 (20)
---Videothoracoscopy and pleurodesis	3 (10)
---Other	1 (3)
Never	9 (30)
**Median age** (years) (range)	64.1 (35–78)
**Performance Status (ECOG):**	
---0	15 (50)
---1	15 (50)
**Sex:**	
---Female	7 (23)
---Male	23 (77)
**Stage at diagnosis (IMIG):**	
---II	9 (30)
---III	8 (27)
---IV	12 (40)
---Other	1 (3)
**Combination of first-line chemotherapy**:	
---Cisplatin and pemetrexed	21 (70)
---Carboplatin and pemetrexed	9 (30)
**Response to first-line treatment:**	
---Stable Disease	15 (50)
Partial Response	15 (30)

All the patients received a combination of a platin compound and pemetrexed as first line of treatment: 21 patients (70%) received cisplatin and pemetrexed, and 9 carboplatin combined with pemetrexed (30%). The median number of cycles of platin and pemetrexed combination administered as first-line therapy was 5.5 (range: 3–11). Response rate was as follows: 15 patients (50%) showed a stability of the disease, and 15 (50%) a PR. All of them showed progression, median TTP was 13 months (range: 6–57.5 months). At the beginning of re-treatment with a pemetrexed-based rechallenge, PS (ECOG) was 1 in 21 patients (70%) and 2 in 9 (30%) (Table 2). For all the patients, pemetrexed-based re-treatment was administered as second-line.

**Table 2:** Efficacy of re-treatment with pemetrexed-based chemotherapy in 30 patients affected by malignant mesothelioma

	**N. (%)**
**N. of patients**	30 (100)
**Regimen of re-treatment**:	
---Pemetrexed	9 (30)
---Cisplatin + pemetrexed	5 (17)
---Carboplatin + pemetrexed	16 (53)
**Response**:	
---Stable disease	15 (50)
---Partial response	5 (17)
---Disease progression	10 (33)
**Performance Status**	
---0	0
---1	21 (70)
---2	9 (30)
**Symptom palliation:**	
---Worsening	10 (33)
---Absence of pain (before treatment and after)	7 (23)
---Pain reduction	13 (43)

Median number of cycles at re-treatment was 4 (range: 3–9). The combination of choice was carboplatin plus pemetrexed in 16 patients (53%), pemetrexed alone was chosen in 9 patients (30%), and 5 patients (17%) received cisplatin and pemetrexed again as they received for first-line.

Modified RECIST criteria were available for 26 patients out of 30; for three patients progression was due to a relapse of pleural effusion and in one patient the retrieval of the radiological images was not possible. Response rate after re-treatment was as follows: 10 patients showed progression of disease (33%), most of patients had stability of disease, 15 out of 30 patients (50%), while 5 patients showed a partial response (17%); median duration of stable disease in patients being retreated was 4.7 months.

**Figure 1:**
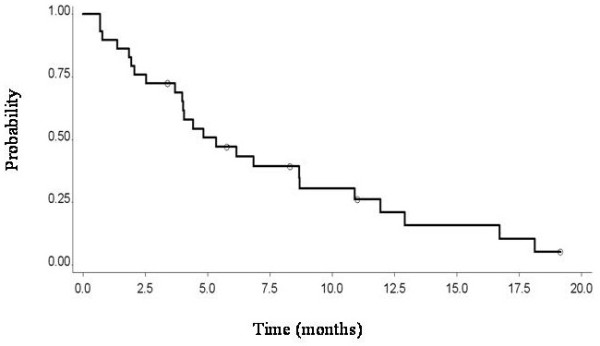
Time to progression of 30 patients affected by mesothelioma and retreated with pemetrexed-based therapy at relapse after first line chemotheraphy.

**Figure 2:**
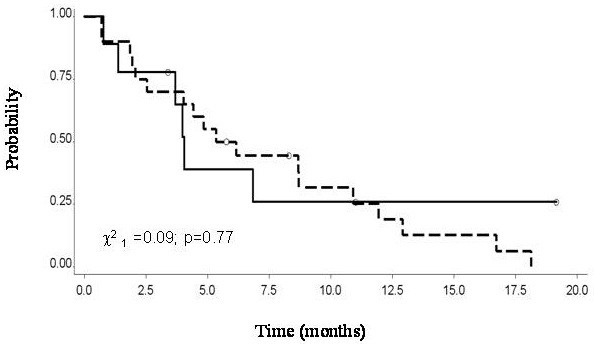
Time to progression of 30 patients with mesothelioma by treatment (__Alimta vs.__Platinum+Alimta).

TTP calculated from the beginning of re-treatment with pemetrexed-based therapy and radiologic progression was 5.1 months (Figure 1). There was no significant difference between TTP of the group of patients receiving pemetrexed combined with platin or pemetrexed alone as re-challenge, 5.7 versus 4 months, respectively (Figure 2).

**Figure 3:**
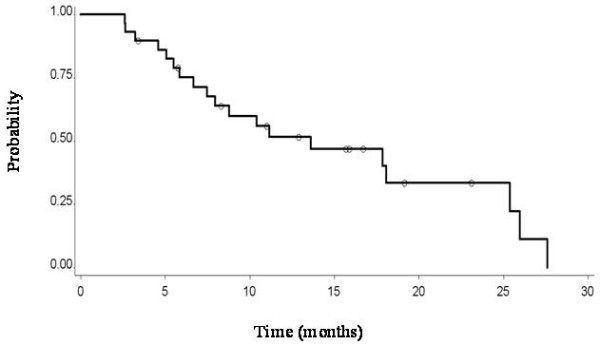
Overall survival of 30 patients affected by mesothelioma and retreated with pemetrexed-based theraph at relapse after first line chemotheraphy.

Median OS, calculated from the beginning of re-treatment to the last contact, was 13.6 months (Figure 3); at the time of analysis at the end of June 2011, 20 patients had died and 10 were still alive.

We also collected information during re-treatment about symptom control, based on VAS patient global assessment [19,20]; all the Institutions were involved in a monitoring program to ensure pain control, therefore VAS administration was part of routine care. Pain decreased in 13 out of 30 patients (43%), 7 patients (23%) had no pain at all, while 10 patients (33%) reported a worsening of their pain.

## Discussion

Malignant mesothelioma is a very aggressive disease with poor outcome even when radical surgery is possible; palliative chemotherapy is offered when patients are not candidate for curative surgery; in randomized clinical trials a platin-combination with pemetrexed or raltitrexed have given better results [4,5]. Out of those combinations, there are no recommended salvage therapies, when patients experience a progression. For other malignancies, when progression occurs at least 6 months after the end of previous treatment, a re-challenge with the same drugs is offered because an antiblastic activity is considered still possible. Based on the lack of alternatives and in analogy with other malignancies, we proposed a re-treatment with the same therapy used as first-line for patients affected by advanced mesothelioma and progressed after obtaining a benefit from first-line combination with platin and pemetrexed. We collected data about 30 patients from several Italian institutions, where the same policy was applied. We found that re-treatment with pemetrexed was feasible in all our patients; a more frequent combination of pemetrexed with carboplatin instead of cisplatin was chosen, likely due to an effort to avoid neurologic or nephrologic toxicities from cisplatin. Once again an interesting response was obtained, with a clinical benefit observed in 20 out of 30 patients (disease control rate, DCR, 66%, PR and SD), both by radiological assessment and reduction of pain. Overall TTP and survival were promising for a second-line setting of patients with advanced mesothelioma, 5.1 and 13.6 months, respectively, considering other similar reports from the literature: a French cohort, where several different drugs were used in second-line for patients affected by mesothelioma, showed 3.8 and 12.2 months, respectively for TTP and OS [21], while in a mono-institutional Italian cohort salvage therapy with gemcitabine-platin combination in second or further line demonstrated 3 and 5.5 months, respectively as TTP and OS [22]. We are aware that our group of patients was selected for good prognostic factors, such as PS, benefit after first-line treatment (SD or RP) and a quite long TTP after the end of first-line treatment; moreover no patient was affected by pure sarcomatoid histotype, considered one of the worst prognostic factors [23,24]. We found a slightly better trend in TTP for patients treated with a platin-pemetrexed combination even at re-treatment; however, the difference was not statistically significant, and could just underline a better PS or younger age at the base of treatment choice.

At the time of preparation of this manuscript it has been just published a similar experience by Ceresoli and Colleagues [25]. They collected data about a similar casistic of patients, 31 patients, treated in a single Institute. Their population was a little different for the inclusion criteria they set up for re-treatment with the same drugs used as first-line, choosing to re-treat with a pemetrexed-based therapy all the patients with a time to progression greater or equal than 3 months after the end of first line. In the Ceresoli’s paper, almost half of patients were re-treated with pemetrexed-combination as second-line, and half as further line of therapy, while all our patients received pemetrexed alone or in combination with a platin compound as second-line therapy only.

The difference in TTP after first-line as inclusion criteria and pemetrexed-based re-treatment used beyond second-line in almost half of the collected patients may explain the shorter TTP and OS observed in Ceresoli’s patients than in ours, 3.8 months and 5.1, respectively, although any significant comparison can be drawn between the two groups of patients.

## Conclusions

Interestingly there are two papers, Ceresoli’s and ours, with similar results. Our patients consistently support the data from Ceresoli and Colleagues and strengthen the results, also for the homogenously use of pemetrexed-based re-treatment as second-line and the multi-institutional collection of data. When patients had a benefit from a platin-pemetrexed combination, in the absence of other effective strategies, we strongly suggest a re-treatment with the same drugs, because an improvement of the symptoms and a consistent gain in TTP can be still obtained.

## Competing interests

The authors declare they have no competing interests.

## Authors’ contributions

AB, GR, AS, VdP, GF, FR, AF, MB, SS and UT were involved in the conduction of therapy. AB drafted the manuscript. RT was responsible for the statistical analysis. All authors contributed to data interpretation and revised the draft critically for scientific content. EB was responsible for data collection. All authors read and approved the final version of the manuscript.
